# Engineering and Evaluation of a Live-Attenuated Vaccine Candidate with Enhanced Type 1 Fimbriae Expression to Optimize Protection Against *Salmonella* Typhimurium

**DOI:** 10.3390/vaccines13060659

**Published:** 2025-06-19

**Authors:** Patricia García, Arianna Rodríguez-Coello, Andrea García-Pose, María Del Carmen Fernández-López, Andrea Muras, Miriam Moscoso, Alejandro Beceiro, Germán Bou

**Affiliations:** 1 Servicio de Microbiología, Instituto de Investigación Biomédica de A Coruña (INIBIC), Complexo Hospitalario Universitario de A Coruña (CHUAC), Sergas, 15006 A Coruña, Spain; patricia.garcia.fernandez@sergas.es (P.G.); arianna.rodriguez.coello@sergas.es (A.R.-C.); andrea.garcia.pose@sergas.es (A.G.-P.); m.a.carmen.fernandez.lopez@sergas.es (M.D.C.F.-L.); andrea.muras.mora@sergas.es (A.M.); mirian.moscoso.naya@sergas.es (M.M.); alejandro.beceiro.casas@sergas.es (A.B.); 2 Centro de Investigación Biomédica en Red de Enfermedades Infecciosas (CIBERINFEC), Instituto de Salud Carlos III, 28029 Madrid, Spain; 3 Departamento de Fisioterapia, Medicina y Ciencias Biomédicas, Universidad de A Coruña, 15006 A Coruña, Spain

**Keywords:** live auxotrophic vaccines, type 1 fimbriae, FimH, mucosal vaccine, fecal IgA, *Salmonella* Typhimurium, intestinal infection model

## Abstract

**Background:***Salmonella* Typhimurium is a major zoonotic pathogen, in which type 1 fimbriae play a crucial role in intestinal colonization and immune modulation. This study aimed to improve the protective immunity of a previously developed growth-deficient strain—a double auxotroph for D-glutamate and D-alanine—by engineering the inducible expression of type 1 fimbriae. **Methods**: P*_tetA_*-driven expression of the *fim* operon was achieved by λ-Red mutagenesis. *fimA* expression was quantified by qRT-PCR, and fimbriation visualized by transmission electron microscopy. Adhesive properties were evaluated through FimH sequence analysis, yeast agglutination, mannose-binding/inhibition assays, and HT-29 cell adherence. BALB/c mice were immunized orogastrically with IRTA ΔΔΔ or IRTA ΔΔΔ P*_tetA_*::*fim*. Safety and immunogenicity were assessed by clinical monitoring, bacterial load, fecal shedding, ELISA tests, and adhesion/blocking assays using fecal extracts. Protection was evaluated after challenging with wild-type and heterologous strains. **Results:** IRTA ΔΔΔ P*_tetA_*::*fim* showed robust *fimA* expression, dense fimbrial coverage, a marked mannose-sensitive adhesive phenotype and enhanced HT-29 attachment. Fimbrial overexpression did not alter intestinal colonization or translocation to mesenteric lymph nodes (mLNs). Immunization elicited a mixed IgG1/IgG2a, significantly increased IgA and IgG against type 1 fimbriae-expressing *Salmonella*, and enhanced the ability of fecal extracts to inhibit the adherence of wild-type strains. Upon challenge (IRTA wild-type/20220258), IRTA ΔΔΔ P*_tetA_*::*fim* reduced infection burden in the cecum (−1.46/1.47-log), large intestine (−1.35/2.17-log), mLNs (−1.32/0.98-log) and systemic organs more effectively than IRTA ΔΔΔ. **Conclusions**: Inducible expression of type 1 fimbriae enhances mucosal immunity and protection, supporting their inclusion in next-generation *Salmonella* vaccines. Future work should assess cross-protection and optimize FimH-mediated targeting for mucosal delivery.

## 1. Introduction

*Salmonella enterica* serovar Typhimurium (*S*. Typhimurium) is an important zoonotic pathogen with global implications. In humans, it is a leading cause of foodborne illnesses, particularly gastroenteritis, and it has also emerged as a major pathogen in invasive infections [1,2]. *S*. Typhimurium also affects livestock and poultry, leading to substantial economic losses in the agricultural sector [3,4]. From a One Health perspective, controlling *S*. Typhimurium is essential, and vaccination offers a decisive solution. In animals, vaccines can reduce disease incidence and pathogen shedding, ultimately decreasing the bacterial load entering the food chain [5]. For humans, particularly immunocompromised individuals or people living in endemic and outbreak-prone regions, targeted vaccines can significantly reduce morbidity and mortality [6]. *S*. Typhimurium initiates infection by adhering to the intestinal mucosa and subsequently invading epithelial cells [7]. Surface structures, such as pili or fimbriae, promote initial interaction with host cell receptors, thereby facilitating bacterial colonization [8]. Type 1 fimbriae are among the most common adhesive organelles in the members of the *Enterobacteriaceae* family, including *Salmonella* species, and are considered a critical virulence factor [9]. Their assembly is mediated by the chaperone–usher pathway, with all biogenesis- and structure-related proteins encoded within a single operon regulated by the *fimA* promoter (P*_fimA_*). The main structural component is FimA, while FimH, a lectin-like adhesin located at the tip of the fimbriae, dictates the interaction through binding to mannose-containing receptors. In particular, FimH exhibits polymorphisms that could affect the ability to adhere to host tissues [9]. In addition, type 1 fimbriae have been shown to contribute to the modulation of the host immune response. Specifically, the fimbriae trigger Toll-like receptor 4 (TLR4)-mediated activation, resulting in the production of proinflammatory cytokines and chemokines that recruit immune cells to the site of infection [10,11,12]. Moreover, type 1 fimbriae facilitate the *Salmonella* glycoprotein 2 (GP2)-dependent transcytotic pathway across M cells, which is an essential step for bacterial uptake and the subsequent initiation of mucosal immune responses, including the production of secretory IgA [13,14]. Hence, type 1 fimbriae have been explored as adjuvants in vaccine formulations targeting *Salmonella* and other priority pathogens, such as uropathogenic *Escherichia coli*, *Klebsiella pneumoniae* and *Acinetobacter baumannii* [15,16,17,18,19,20,21,22,23,24,25,26,27,28,29,30]. The oral route is suitable for eliciting broad protection owing to the gastrointestinal tropism of *S*. Typhimurium. Indeed, live attenuated bacteria have been widely explored as mucosal vaccines [31,32]. In this context, we previously developed a live *S*. Typhimurium vaccine candidate exhibiting dual auxotrophy for peptidoglycan D-amino acids, specifically D-glutamate and D-alanine, by deleting the genes encoding the glutamate (*murI*) and alanine (*alr* and *dadX*) racemases (IRTA ΔΔΔ) [33]. This prototype vaccine had a favorable safety profile and conferred moderate protection against disease in orally inoculated mice. In the present study, with the aim of improving protective immunity, we genetically engineered the double auxotroph to enable the inducible expression of type 1 fimbriae, and we evaluated the immunogenicity and efficacy of the modified vaccine candidate in a mouse intestinal model.

## 2. Materials and Methods

### 2.1. Bacterial Strains and Growth Conditions

*S*. Typhimurium strains used in this study (Table 1) were grown in Luria–Bertani broth (LB: 10 g/L tryptone, 5 g/L yeast extract, 10 g/L sodium chloride) or on LB agar at 37 °C, unless otherwise stated. If necessary, ampicillin (100 µg/mL) or kanamycin (200 µg/mL; Merck Life Science S.L.U., Madrid, Spain) were used. L-(+)-arabinose, D-glutamate and D-alanine (Sigma-Aldrich) were added at 10 mM, unless otherwise specified. For the induction of *fim* expression, the P*_tetA_*::*fim* derivative was cultured under agitation for 3 h with 100 ng/mL of anhydrotetracycline (ATc, Merck Life Science S.L.U.), unless another concentration is stated. When necessary, *S*. Typhimurium wild-type strains and IRTA ΔΔΔ were grown statically for 18 to 24 h with serial passage (up to the 3rd passage), as these conditions promote fimbrial expression, particularly type 1 fimbriae [34].

### 2.2. Construction of the Fim-Inducible S. Typhimurium Auxotrophic Derivative

The putative promoter sequence of the *fim* operon was replaced on the chromosome of IRTA ΔΔΔ^kanS^ by a gene cassette (*aph(3)-IIIa tetR* P*_tetA_*) amplified from the plasmid p3773 [35]. Primers containing 40 nt extension homologues to the target region were used for λ-Red recombination, facilitated by pKD46 (Table S1, Figure S1) [36]. PCR and sequencing were used to verify the substitution in the derivative IRTA ΔΔΔ P*_tetA_*::*fim*.

### 2.3. Comparison of FimH Amino Acid Sequences

The amino acid sequence of FimH from IRTA GN-3728 (FimH^IRTA GN-3728^) was compared with another 35 full-sequenced *S*. Typhimurium genomes obtained from the BioCyc Genome Database Collection and the NCBI RefSeq database. When necessary, raw reads were assembled (Unicycler v0.4.8) and annotated (RAST toolkit through BV-BRC v3.47.11). The amino acid sequences were aligned using MUSCLE v3.8.425 and visualized in AliView v1.30 for comparison.

### 2.4. RNA Isolation and qRT-PCR

Total RNA was extracted from bacterial cultures using the High Pure RNA Isolation Kit (Roche, Basel, Switzerland), treated with DNAse I (Merck Life Science S.L.U.) and purified with the RNeasy MinElute Cleanup Kit (Qiagen, Hilden, Germany). It was then included as a template in a LightCycler 480 RNA instrument (Roche) using the RNA Master Hydrolysis Probe kit (Roche) with the UPL probes and primers listed in Table S1. The *fimA* gene was quantified at least in triplicate and normalized to transcription levels of *rpoD* housekeeping gene.

### 2.5. Transmission Electron Microscopy (TEM)

Samples were prepared as previously described, with some modifications [35]. Cultures were fixed with 2.5% glutaraldehyde for 30 min, pellets were washed three times in filtered MilliQ water, and 2 µL aliquots were placed for 1 min on formvar/carbon-coated TEM grids to allow adsorption. The grids were negatively stained with 0.5% uranyl acetate for 30 s, blotted with filter paper and air-dried. Samples were observed with transmission electron microscopy (JEOL JEM 1010 electron microscope, 80 kV, JEOL (Europe) BV, Zaventem, Belgium) to determine the degree of fimbriation.

### 2.6. Yeast Agglutination Assay

The mannose-binding phenotype was tested for the ability to agglutinate mannan-containing yeast cells (*Candida albicans* 423 15910, CHUAC) by mixing a yeast suspension with serial dilutions of bacterial cultures on glass slides [37]. Positive agglutination was visually assessed after incubation of the slides for 5 min with orbital rotation at 100 rpm.

### 2.7. D-Mannose Binding and Blocking Assays

For binding/blocking assays, 96-well microtiter plates (Nunc high-binding, Thermo Fisher Scientific, S.L., Madrid, España) were coated with 20 µg/mL of D-mannose-BSA (NGP1108, Dextra, Reading, UK) or Bovine Serum Albumin (BSA, Merck Life Science S.L.U.) alone in 50 mM carbonate/bicarbonate buffer (pH 9.5) at 37 °C for 2 h [35]. Excess protein was removed by washing with phosphate-buffered saline (PBS), and non-specific binding was minimized by incubating with BSA (20 mg/mL, 37 °C for 2 h). For adhesion, 100 µL of bacterial suspension (5 × 10^8^ CFU/mL) was added to each well, and the plates were incubated at 37 °C for 1 h. To check the D-mannose binding specificity, bacteria were incubated in the presence of 1% of monosaccharide 4-Aminophenil α-D-mannopyranoside (Sigma) or without soluble inhibitors on BSA-coated wells [38]. Microphotographs were taken in an inverted Fluorescence Microscope (Nikon, Eclipse Ti, Nikon Europe B.V, Amstelveen, The Netherlands) in optical mode. Bacterial density was quantified after crystal violet staining (0.04% for 20 min) and subsequent solubilization in 30% acetic acid. Optical density was measured at 590nm (OD_590_) in a NanoQuant infinite M200 Pro spectrophotometer (Tecan Group Ltd., Männedorf, Switzerland). All experiments were carried out in triplicate.

### 2.8. Bacterial Adhesion to HT-29 Human Colorectal Cells and Inhibition Assays

HT-29 cells were routinely grown in the presence of 5% CO_2_ at 37 °C, on McCoy’s 5A Medium 1× (Gibco, Thermo Fisher Scientific, S.L.) supplemented with 10% Fetal Bovine Serum (FBS), 1% Penicillin/Streptomycin and 1% Glutamax (Gibco, Thermo Fisher Scientific, S.L.). Bacteria, adjusted to an OD_600_ of 1, were recovered by centrifugation at 4100× *g* for 10 min, washed twice with sterile saline (0.9% NaCl solution) and suspended at 10^8^ CFU/mL in HBSS 1×. For inhibition assays, the cells were pre-incubated with 1% of the monosaccharide 4-Aminophenil α-D-mannopyranoside or with pooled fecal extracts. Adhesion was evaluated on washed 5 × 10^5^ HT-29 monolayers, plated at a multiplicity of infection (MOI) of 100, in 24-well plates (Corning Costar TC-Treated Plates, Corning, Thermo Fisher Scientific, S.L.) incubated for 30 min at 37 °C/5% CO_2_. Non-adhered bacteria were removed by washing twice with saline, and 0.5% sodium deoxycholate (Merck Life Science S.L.U.) was added to the wells for 10 min at 37 °C. Serial 10-fold dilutions were prepared in saline solution and plated onto LB agar plates and incubated at 37 °C for 24 h. Adhesion was expressed as the percentage number of adhered bacteria relative to the total number of bacteria used in the experiment. In blocking experiments, adhesion was expressed as a percentage relative to the value obtained for the fecal extracts from the control mice administered saline.

### 2.9. Mouse Immunization, Sampling and Challenge Experiments

Female BALB/c mice (*n* = 70), aged 7–10 weeks, were used and housed in cages with no more than five animals per cage. All mice were bred and maintained under specific pathogen-free conditions in the Centro Tecnológico de Formación de la Xerencia de Xestión Integrada A Coruña (CTF-XXIAC) of the Galician Health Service (SERGAS). Immunization was conducted by administration of three orogastric doses, at 14-day intervals, with approximately 10^9^ CFU of IRTA ΔΔΔ, IRTA ΔΔΔ P*_tetA_*::*fim* or saline (controls) (*n* = 9 per group). Mice were weighed and monitored for clinical signs of disease, including fur piloerection, hunched posture, diarrhea, lethargy, and abnormal behavior or mobility. Fecal shedding was quantified by CFU enumeration, and blood and fecal samples were collected and processed for ELISA tests as previously described [33]. For safety determinations exclusively, mice inoculated with vaccine strains (*n* = 8 per group) were euthanized at different times, and the bacterial burden was determined in the cecum, large and small intestines, and mLNs. Infections with IRTA GN-3728 (wild-type) and 20220258 (heterologous) strains were induced on days 48 and 56, respectively, in mice pre-treated with 20 mg of streptomycin 24 h before inoculation with bacteria. Mice were monitored daily for clinical signs of disease and survival until euthanasia at day 8 post infection (dpi). Cecum, large intestine, mLNs, spleen and liver were aseptically recovered, homogenized and plated on *Salmonella* chromogenic agar (Condalab, Madrid, Spain) to determine CFU/g. The researchers were not blinded to the procedure.

### 2.10. ELISA Assays

The levels of specific antibodies in fecal and serum samples were measured by ELISA against formalin-inactivated IRTA GN-3728 (agitation culture) or IRTA ΔΔΔ P*_tetA_*::*fim* (with ATc), as previously reported [33]. Briefly, bacteria were inactivated by incubation with 1% (*v*/*v*) paraformaldehyde and used to coat 96-well high-binding plates (Nunc MaxiSorp, Thermo Fisher Scientific, S.L.) at 4 °C overnight. Plates were then washed five times with PBS and blocked with 5% skim milk in PBS for 1 h at 37 °C. After five additional washes with 0.005% Tween 20 in PBS, serial dilutions of mouse samples prepared in DMEM supplemented with 10% FBS were added and incubated overnight at 4 °C. Subsequently, plates were washed again, and 100 µL of HRP-conjugated anti-mouse secondary antibodies diluted 1:5000 in DMEM with 10% FBS was added to detect total IgG and its subtypes (Merck Life Science S.L.U.) or IgA (Bethyl Laboratories Inc., Boston, Massachusetts, USA). The reaction was developed by adding 100 µL of 3,3′,5,5′-Tetramethylbenzidine (Merck Life Science S.L.U.) per well, and stopped with 50 μL of 1 M H_2_SO_4_. Absorbance was read at OD_450_. The endpoint titer was defined as the highest sample dilution with an absorbance value at least 0.1 values above the blank.

### 2.11. Statistical Analyses

All statistical comparisons were conducted in GraphPad Prism 5 (GraphPad Software v6.01, Inc., San Diego, CA, USA). Student’s *t* test (Welch’s correction) was used to compare mean antibody titers and percentage adhesion. The Mann–Whitney *U* test was used to compare means of bacterial loads between pairs of mice groups. A value of *p* < 0.05 was considered statistically significant.

## 3. Results

### 3.1. Construction and Characterization of a Double Auxotrophic Derivative Expressing Type 1 Fimbriae in S. Typhimurium

**Figure 1:**
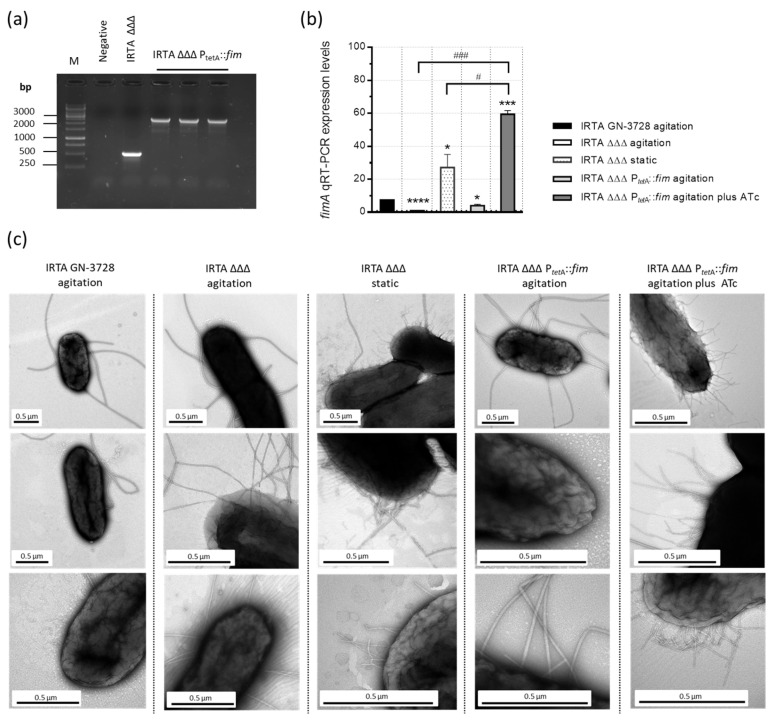
Construction and verification of *S*. Typhimurium IRTA ΔΔΔ P*_tetA_*::*fim* auxotrophic derivative with inducible expression of type 1 fimbriae. (**a**) PCR verification of *aph(3′)-IIIa tetR* P*_tetA_* cassette integration in the native *fimA* promoter region. The amplicon sizes are 535 bp (IRTA ΔΔΔ) and 2663 bp (IRTA ΔΔΔ P*_tetA_*::*fim*). Lane M, GeneRuler 1 Kb Plus DNA Ladder. A detailed schematic representation of the mutagenesis protocol is depicted in Figure S1. (**b**) Expression levels of the *fimA* gene, determined by qRT-PCR (mean ± SD; *n* = 3 biological replicates) in IRTA GN-3728 and its auxotrophic derivatives obtained under different culture conditions, and normalized to the *rpoD* reference gene. Primers and UPL probes used are listed in Table S1. Student’s *t*-test (Welch’s correction): * *p* < 0.05, *** *p* < 0.005, **** *p* < 0.0001, relative to IRTA GN-3728, and ^#^ *p* < 0.05, ^###^ *p* < 0.005 between the indicated groups. (**c**) TEM micrographs of fimbriation patterns. Scale bar: 0.5 µm. Static, 3rd serial passage without agitation; ATc, 100 ng/mL.

Genomic analysis of the IRTA GN-3728 revealed the presence of an intact operon for Fim biogenesis. To obtain the IRTA ΔΔΔ P*_tetA_*::*fim* derivative, a fragment of 139 bp located upstream of the *fimA* gene was replaced with a gene cassette from p3773, in IRTA ΔΔΔ^kanS^ [33,35]. Successful integration of the cassette in IRTA ΔΔΔ P*_tetA_*::*fim* was confirmed by PCR amplification (Figure 1a) and sequencing of the recombinant PCR fragment.

In addition, the optimal inducer concentration of ATc was determined by qRT-PCR analysis of the *fimA* gene (Figure S2). Comparative analysis of the *fimA* expression was also performed. As shown in Figure 1b, *fimA* expression was significantly enhanced in the IRTA ΔΔΔ P*_tetA_*::*fim* strain cultured under agitation in the presence of ATc, resulting in a remarkable 62-fold increase relative to IRTA ΔΔΔ. Notably, the deletion of racemase genes in IRTA ΔΔΔ led to a 7.8-fold reduction in *fimA* expression compared to IRTA GN-3728 but increased after being grown under static serial passages, as these conditions promote type 1 fimbriae [34]. Accordingly, fimbriae were not detected in the wild-type, IRTA ΔΔΔ or IRTA ΔΔΔ P*_tetA_*::*fim* (without ATc) strains when grown under agitation, as these strains remained mostly non-fimbriated (Figure 1c). By contrast, peritrichous appendages were observed on the surface of IRTA ΔΔΔ P*_tetA_*::*fim* and IRTA ΔΔΔ after induction with ATc or repeated subculture under static conditions, respectively. Moreover, fimbriae overexpression did not affect bacterial viability or cell morphology.

**Figure 2:**
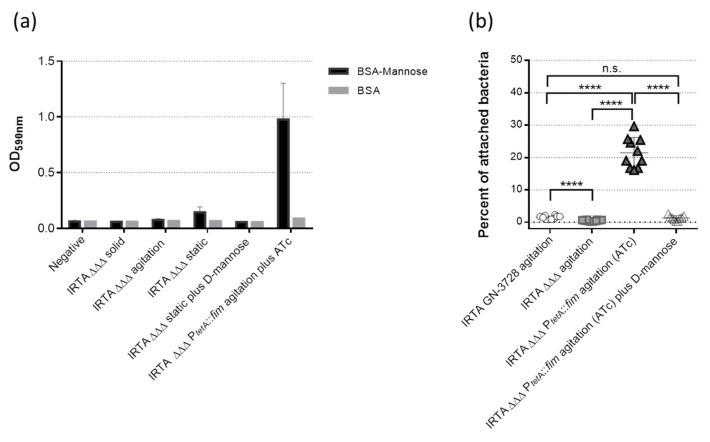
Characterization of *S*. Typhimurium IRTA ΔΔΔ P*_tetA_*::*fim* auxotrophic derivative. (**a**) In vitro binding to immobilized BSA-mannose measured as absorbance (mean ± SD) following crystal violet staining. (**b**) Percentage of bacterial adherence to HT-29 cells (mean ± SD). **** *p* < 0.0001, Student’s *t*-test (Welch’s correction). n.s., not significant. D-mannose: 4-Aminophenil α-D-mannopyranoside.

Functional analysis was also carried out. The adhesive phenotype was confirmed by its ability to agglutinate yeast cells (Figure S3) and to specifically bind to mannose-BSA-coated surfaces (Figure S4 and Figure 2a). In particular, after induction with ATc, IRTA ΔΔΔ P*_tetA_*::*fim* exhibited strong binding to mannose-BSA. The level of binding was reduced in the presence of the 4-Aminophenyl α-D-mannopyranoside and was not observed with BSA alone, demonstrating mannose-dependent adhesion. Consistent with previous findings (qRT-PCR and TEM), the IRTA ΔΔΔ strain grown under agitation showed limited interaction with mannose-BSA.

Furthermore, the adhesion capacity of IRTA wild-type, IRTA ΔΔΔ and IRTA ΔΔΔ P*_tetA_*::*fim* strains were evaluated in human HT-29 cell monolayers (Figure 2b). As expected, IRTA ΔΔΔ showed significantly lower attachment than IRTA wild-type, while IRTA ΔΔΔ P*_tetA_*::*fim* grown with ATc exhibited the highest level of adherence. The enhanced attachment was abolished in the presence of 4-Aminophenil α-D-mannopyranoside, which further supports mannose-sensitive interaction.

Finally, a comprehensive sequence analysis of *S*. Typhimurium FimH proteins was conducted, given that certain polymorphisms may affect their adhesion capacity. The analysis revealed that FimH^IRTA GN−3728^ shares an amino acid sequence with a previously described FimH^SL1344^ low-binding variant [39], except for four substitutions: Q89R, L126R and Y131S in the lectin domain, and I317N in the pilin domain (Figure S5).

### 3.2. Safety Profile and Humoral Immune Responses to Vaccination

To evaluate vaccine safety, the bacterial burden was assessed in the small and large intestines, cecum and mLNs of mice inoculated with 10^9^ CFUs of IRTA ΔΔΔ and IRTA ΔΔΔ P*_tetA_*::*fim* strains (Figure S6). No significant differences in bacterial loads were observed at early time points, except in the large intestine and cecum at 2 h post-inoculation. Moreover, both strains exhibited similar levels of mLNs colonization and fecal shedding, and no adverse effects on appearance, behavior or body weight were observed throughout the immunization experiments, which consisted of three repeated doses of 10^9^ CFUs (Figure S6), confirming the safety profile of the IRTA ΔΔΔ P*_tetA_*::*fim* derivative. Importantly, a high dose of 10^9^ CFUs, commonly used in oral live-attenuated vaccine regimens, did not result in any mortality, even after repeated administrations.

**Figure 3:**
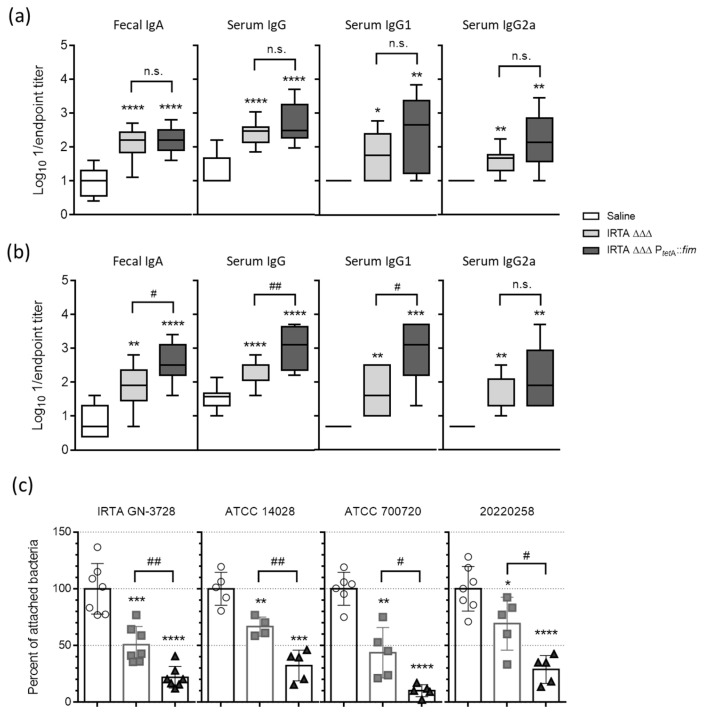
Comparison of humoral immune responses induced by *S*. Typhimurium auxotrophic derivatives. (**a**,**b**) Log_10_ 1/Endpoint titer of fecal IgA and serum IgG, IgG1 and IgG2a antibodies produced by BALB/c mice on day 47 post-vaccination. ELISA plates were coated with (**a**) IRTA GN-3728 or (**b**) IRTA ΔΔΔ P*_tetA_*::*fim*. Antibody responses were measured in mice immunized with three doses of 10^9^ CFU of IRTA ΔΔΔ or IRTA ΔΔΔ P*_tetA_*::*fim*, as well as in control (saline-treated) mice. Each experimental group consisted of 6–9 mice. (**c**) Percentage adherence of the indicated *S*. Typhimurium strains to HT-29 cells (mean ± SD) after pre-incubation with fecal extracts from control mice injected with saline (open circles) or mice immunized with IRTA ΔΔΔ (gray squares) or IRTA ΔΔΔ P*_tetA_*::*fim* (black triangles). Data are expressed relative to adhesion after pre-incubation with fecal extracts from control mice (100% reference value). Student’s *t*-test with Welch’s correction: * *p* < 0.05, ** *p* < 0.005, *** *p* < 0.0005, **** *p* < 0.0001, relative to control mice, or ^#^ *p* < 0.05, ^##^ *p* < 0.005 between vaccinated groups. n.s., Not significant.

On day 47 post vaccination, statistically significantly higher levels of IgA, IgG and their subtypes were observed in all mice administered IRTA ΔΔΔ and IRTA ΔΔΔ P*_tetA_*::*fim* compared to the control mice, with an IgG1/IgG2a ratio approaching 1. Notably, no significant differences were observed in antibody titers in the two vaccinated groups when tested against IRTA wild type (Figure 3a). However, fecal IgA and also serum IgG and IgG1 only increased in the IRTA ΔΔΔ P*_tetA_*::*fim* group when the high-fimbriated bacteria were used for coating. These titers were significantly higher than those in the IRTA ΔΔΔ group, suggesting the presence of specific antibodies against type 1 fimbriae in mice immunized with IRTA ΔΔΔ P*_tetA_*::*fim* (Figure 3b). We therefore investigated whether fecal extracts could inhibit the adhesion of wild-type *S*. Typhimurium to intestinal HT-29 cells. First, the attachment of selected wild-type strains was assessed against HT-29 monolayers (Figure S7). Subsequently, bacterial adhesion was inhibited by pre-incubation with fecal samples from immunized mice, with the highest inhibition observed in samples from mice immunized with IRTA ΔΔΔ P*_tetA_*::*fim* (Figure 3c).

### 3.3. Protective Efficacy in Vaccinated BALB/c Mice Against Virulent Challenge

**Figure 4:**
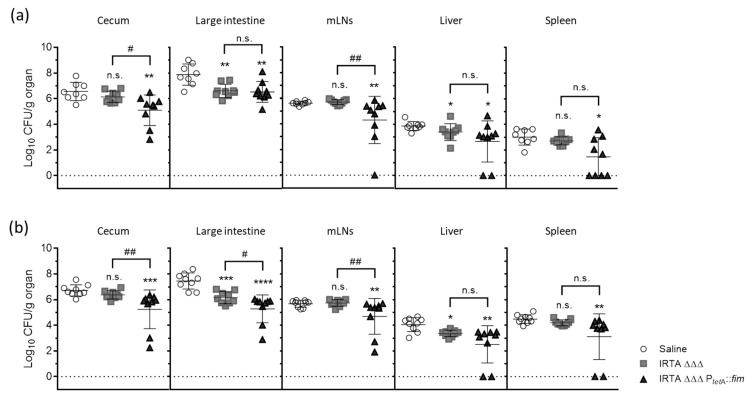
Comparison of protective efficacy mediated by *S*. Typhimurium auxotrophic derivatives. Bacterial load (Log_10_ CFU/g) in organs recovered from immunized and control (administered saline) mice, after infection with (**a**) IRTA GN-3728 (4 × 10^6^ CFU) and (**b**) 20220258 (2 × 10^5^ CFU). Mann–Whitney U test: * *p* < 0.05, ** *p* < 0.005, *** *p* < 0.0005, **** *p* < 0.0001, relative to control mice, or ^#^ *p* < 0.05, ^##^ *p* < 0.005 between vaccinated groups. n.s., Not significant.

Mice immunized with three doses (10^9^ CFUs) of the candidate vaccine were challenged with *S*. Typhimurium wild-type strains, and the bacterial burden was assessed on dpi 8 (Figure 4).

Infection with both IRTA GN-3728 and 20220258 strains resulted in consistent bacterial loads in the cecum, large intestine, mLNs and extraintestinal tissues, including the liver and spleen, in control mice. Additionally, one mouse in the control group succumbed on dpi 2, highlighting the severity of IRTA GN-3728 infection. In mice inoculated with IRTA ΔΔΔ, the bacterial loads in the large intestine and liver were significantly lower than in the control mice. The average reductions were 1.27/1.30 log-units in the large intestine and 0.47/0.69 log-units in the liver, following infection with IRTA GN-3728/20220258 strains, respectively. However, no reduction was observed in other tissues analyzed. By contrast, the administration of IRTA ΔΔΔ P*_tetA_*::*fim* resulted in significant protection across all tissues, including the cecum, mLNs and spleen. After infection with IRTA GN-3728/20220258 strains, the average reductions, relative to control mice, were 1.46/1.47 log-units in the cecum, 1.35/2.17 log-units in the large intestine and 1.32/0.98 log-units in mLNs, respectively. Moreover, in several of the mice, colonization was not detected in the liver or spleen, which suggests that immunization with the IRTA ΔΔΔ P*_tetA_*::*fim* candidate limited infection more extensively, including against the heterologous strain 20220258, compared to immunization with the IRTA ΔΔΔ strain.

## 4. Discussion

*S*. Typhimurium is a prevalent zoonotic agent and its widespread presence in humans and animals constitutes a public health concern, highlighting the need for effective control strategies [40]. Although vaccination is a key strategy, current licensed vaccines are limited to livestock and have variable efficacy [5]. Live-attenuated candidate vaccines are particularly promising owing to their ability to induce robust cellular and mucosal immunity. Nevertheless, safety concerns have been raised, as these replication-competent strains are associated with prolonged colonization and persistent shedding [41,42], and they are not recommended for use in infants or immunocompromised individuals. In an effort to circumvent this limitation, we previously engineered a live but fully growth-deficient *S*. Typhimurium vaccine candidate (IRTA ΔΔΔ) [33], which exhibited minimal mucosal inflammation, transient shedding and no long-term invasiveness in mice. Despite inducing moderate mucosal IgA and serum IgG responses, the protective efficacy of the candidate was relatively low. A good balance between attenuation and immunogenicity is crucial in mucosal vaccination, as excessive attenuation may impair the ability to reach inductive sites and ultimately reduce the protective efficacy [43]. In this study, we aimed to address this challenge by exploiting the advantages offered by type 1 fimbriae, naturally encoded by *S*. Typhimurium, to improve the protective immunity of IRTA ΔΔΔ. The new vaccine strain, IRTA ΔΔΔ P*_tetA_*::*fim*, exhibited an enhanced expression of the *fim* operon, encoding type 1 fimbriae that densely cover the bacterial surface. It also displayed a marked mannose-sensitive adhesive phenotype with improved attachment to HT-29 cell monolayers. Importantly, type 1 fimbriae can enhance mucosal immunity by increasing bacterial adherence to intestinal surfaces, prolonging antigen availability and facilitating interaction with mucosal immune cells to initiate effective immune responses. Variants of the lectin-like adhesin have been identified in *S*. Typhimurium, with potential implications for altered binding specificity and affinity to host cell receptors [9,39]. Specifically, FimH^IRTA GN-3728^ harbors G61 and F118 in the lectin domain, consistent with a known low-binding variant. Interestingly, four amino acid substitutions (Q89R, L126R, Y131S and I317N) were identified, and functional studies will be necessary to elucidate their biological relevance and potential impact on FimH-mediated adhesion. This study found no substantial differences between IRTA ΔΔΔ and IRTA ΔΔΔ P*_tetA_*::*fim* derivatives regarding their capacity to transiently colonize intestinal tissues or to translocate as viable bacteria to the mLNs in the mouse model, and both strains exhibited comparable safety profiles. In particular, no adverse effects or mortality were observed, even at high doses (10^9^ CFUs) and after repeated administrations. These findings are consistent with the expected behavior of auxotrophic vaccine candidates, which exhibit self-limiting growth in vivo [44], and suggest that type 1 fimbriae expression does not enhance *S*. Typhimurium virulence in the auxotrophic background. In contrast to many previously tested strains that rely on mutations that carry residual risks of reversion or systemic dissemination [41,42,45,46,47,48,49,50,51], our vaccine platform utilizes double auxotrophy for D-glutamate and D-alanine, achieved through the complete deletion of three racemase-encoding genes, which confers a high degree of genetic and phenotypic stability, resulting in a robust safety profile [33]. Although FimH has been shown to facilitate M cell targeting and improve oral vaccine uptake [14,26,52], the present study was limited to viable bacteria, and we cannot rule out the possibility of increased translocation of non-viable bacteria and/or bacterial antigens. Therefore, it remains to be determined whether fimbriae promote M cell uptake in this context. In addition, site-directed mutagenesis could be used to generate FimH variants with tailor-made binding properties, offering opportunities for rational mucosal vaccine design. Notably, type 1 fimbriae have been identified as potential vaccine targets through in silico approaches [53,54,55,56,57], and their use in bacterial vaccine development has yielded promising immunological outcomes in preclinical models [16,17,19,20,21,23,24,25,27,28,29,30]. Our findings demonstrate that IRTA auxotrophic derivatives induce strong mucosal and systemic immunity, with a mixed Th1/Th2 response, which is critical for both cellular and humoral pathogen clearance [58]. Although both vaccine strains induced comparable antibody responses to non-fimbriated bacteria (likely reflecting a recognition of conserved core antigens), only IRTA ΔΔΔ P*_tetA_*::*fim* triggered significantly higher titers against type 1-expressing bacteria and demonstrated an enhanced inhibition of wild-type *S*. Typhimurium adhesion to HT-29 intestinal cells, as shown by activity in fecal extracts. These results are consistent with those of previous studies showing that IgA targeting bacterial surface structures, such as fimbriae, can effectively block bacterial colonization by disrupting key pathogen–host interactions [59,60,61,62,63]. Consequently, while IRTA ΔΔΔ primarily provides protection in the large intestine and liver, IRTA ΔΔΔ P*_tetA_*::*fim* conferred broader protection across all tissues evaluated, including the cecum, mLNs and spleen, with undetectable colonization in some mice. When compared with other live-attenuated *S.* Typhimurium vaccine candidates, IRTA ΔΔΔ P*_tetA_*::*fim* demonstrates a favorable balance between safety and efficacy. While some highly immunogenic strains may confer higher levels of protection, this is often achieved at the expense of safety, or depends on metabolic pathways that are less suitable for human applications [45,46,47,48,49,50,51]. Although our candidate did not achieve complete pathogen clearance in all animals, it represents a safer alternative with clear potential for further optimization. Moreover, the phase-variable expression of type 1 fimbriae, often down-regulated in wild-type cultures, may reduce piliated bacteria in the inoculum, underestimating the true contribution of fimbria-specific immune responses in the animal model. Therefore, challenge models that mimic natural infection should be considered to fully exploit the immunoprotective potential of type 1 fimbriae.

## 5. Conclusions

In conclusion, altogether, the study findings support the inclusion of fimbrial antigens in *Salmonella* vaccine design as a means of enhancing mucosal immunity and interfering with critical steps in the infection process. Future studies should investigate the long-term dynamics of immune response as well as protection conferred against diverse fimbrial variants to further validate the translational potential of this strategy in *Salmonella* vaccine platforms.

## Figures and Tables

**Table 1:** *S*. Typhimurium strains used in the study.

Strain	Relevant Features	Experimental Use	Source
IRTA GN-3728	Wild-type rif-resistant	PCR, qRT-PCR, TEM and HT-29 adherence FimH sequence analysis ELISA tests Blocking HT-29 adhesion assays Mouse challenge	[33]
IRTA ΔΔΔ^kanS^	Double auxotroph (Δ*murI* Δ*alr* Δ*dadX*)	λ-Red Mutagenesis	[33]
IRTA ΔΔΔ (formerly IRTA ΔΔΔ::*aph(3)-IIIa*)	Double auxotroph kan-resistant (Δ*murI* Δ*alr*:: *aph(3)-IIIa* Δ*dadX)*	PCR, qRT-PCR, mannose-binding, TEM and HT-29 adherence Mouse immunization	[33]
IRTA ΔΔΔ P*_tetA_*::*fim*	Double auxotroph with inducible *fim* expression, kan-resistant (Δ*murI* Δ*alr* Δ*dadX aph(3)-IIIa-tetR-*P*_tetA_*::*fim*)	PCR, qRT-PCR, mannose-binding, TEM and HT-29 adherence ELISA tests Mouse immunization	This study
ATCC 700720 (LT2)	Wild-type isolated from a natural source	Blocking HT-29 adhesion assays	ATCC
ATCC 14028	Reference strain, isolated from pools of heart and liver from 4-week-old-chickens	Blocking HT-29 adhesion assays	ATCC
20220258 (monophasic)	Multi-country outbreak strain isolated from 38 year-old woman, cgMLST cluster 1, ST34 and amp-, chl-, str-, kan-, gen-, smx-, tet-and tmp-resistant	Blocking HT-29 adhesion assays Mouse challenge	[33]

IRTA, Instituto de Investigación y Tecnología Agroalimentarias, Generalitat de Cataluña; cgMLST, core genome multilocus sequence typing; ST, sequence type; amp, ampicillin; chl, chloramphenicol; str, streptomycin; kan, kanamycin; gen, gentamycin; smx, sulfamethoxazole; rif, rifamycin; tet, tetracycline; tmp, trimethoprim.

## Data Availability

The data presented in this study are available in this article (and Supplementary Material).

## Supplementary Materials

The following supporting information can be downloaded at https://www.mdpi.com/article/10.3390/vaccines13060659/s1, Figure S1: Strategy for the generation of the IRTA ΔΔΔ P*_tetA_*::*fim* derivative strain using the λ-Red recombination system; Figure S2: Expression of the *fimA* gene (mean ± SD) in *S*. Typhimurium IRTA ΔΔΔ P*_tetA_*::*fim* induced by increasing concentrations of ATc; Figure S3: Yeast agglutination activity of *S*. Typhimurium auxotrophic derivatives under specified culture conditions; Figure S4: Representative microscopic images of in vitro binding to immobilized BSA-mannose or BSA alone; Figure S5: Comparison of FimH amino acid sequences from *S*. Typhimurium; Figure S6: Safety profiles of *S*. Typhimurium auxotrophic derivatives; Figure S7: Percentage bacterial adherence to HT-29 colorectal cells obtained from *S*. Typhimurium cultures after growing under conditions inducing type 1 fimbriae; Table S1: Plasmids and primers used in this study.
