# Supplementary material for: Engineering and Evaluation of a Live-Attenuated Vaccine Candidate with Enhanced Type 1 Fimbriae Expression to Optimize Protection Against Salmonella Typhimurium
Source: Vaccines (Basel). 2025 Jun 19;13(6):659. doi: 10.3390/vaccines13060659 (PMC12197609; doi:10.3390/vaccines13060659)
Supplement: Supplementary file 1 [file vaccines-13-00659-s001.zip › vaccines-3684754-supplementary.pdf]

Supplementary information for "Engineering and evaluation of a live-attenuated vaccine candidate with enhanced type 1 expression to optimize protection against *Salmonella* Typhimurium"

by García *et al*.

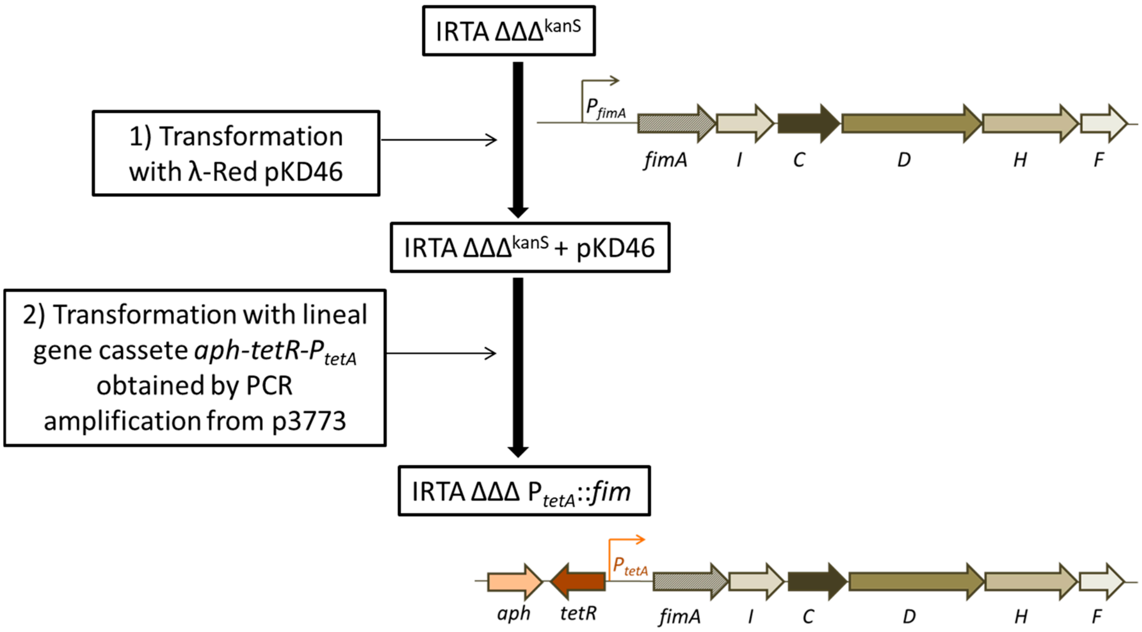

Figure S1. Strategy for the generation of the IRTA ΔΔΔ P*tetA*::*fim* derivative strain using the λ-Red recombination system. The IRTA ΔΔΔ^{kanS} strain, containing an intact *fim* operon, was first transformed with plasmid pKD46, which encodes the λ-Red recombinase genes (*exo*, *bet*, *gam*) under the control of an arabinose-inducible promoter. A linear PCR product carrying an inducible expression cassette [*aph(3')-IIIa*-tetR-P*tetA*] was amplified from the plasmid p3773, using primers containing homology arms to the promoter region of the *fim* operon (Table S1). This fragment was electroporated into the IRTA ΔΔΔ^{kanS} strain harboring pKD46, and recombinants were selected on kanamycin-containing plates, resulting in IRTA ΔΔΔ P*tetA*::*fim* mutant derivative. This procedure was performed following the protocol described by Datsenko and Wanner (2000) and Hansmeier et al. (2017).

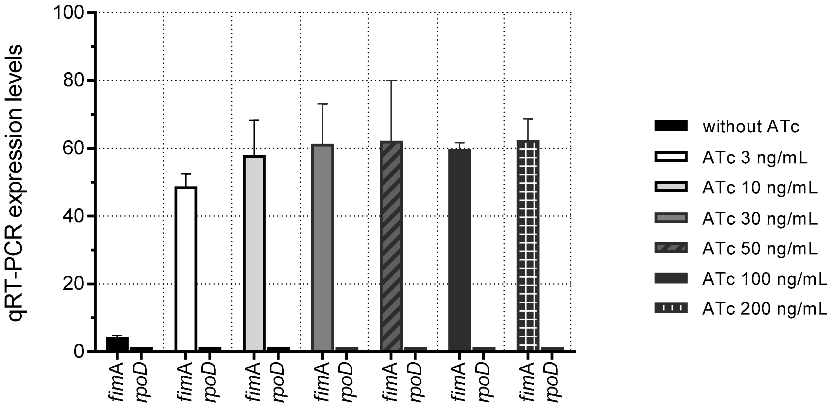

Figure S2. Expression of the *fimA* gene (mean ± SD) in *S*. Typhimurium IRTA ΔΔΔ P_{tetA}::*fim* induced by increasing concentrations of ATc (0-200 ng/mL), determined by qRT-PCR and normalized to the *rpoD* reference gene (value of 1). Primers and UPL-probes used are listed in **Table S1.**

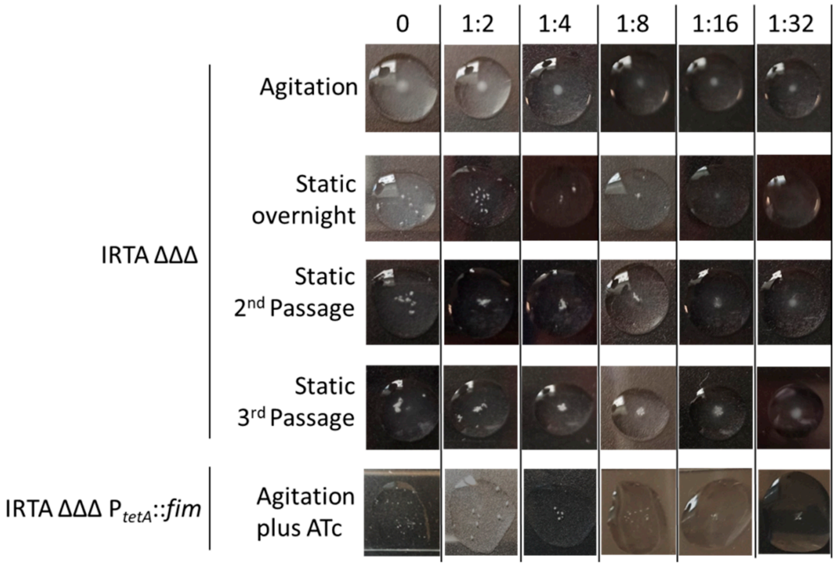

Figure S3. Yeast agglutination activity of *S*. Typhimurium auxotrophic derivatives under specified culture conditions. Serial dilutions (up to 1:32) of bacterial cultures were mixed with equal volumes of *Candida albicans* strain 42315910 (CHUAC), and macroscopic agglutination was visually assessed.

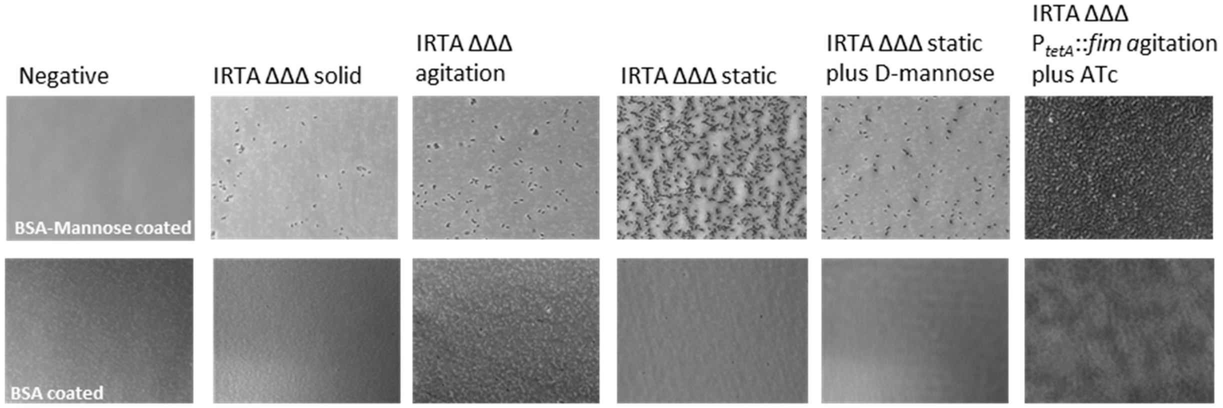

Figure S4. Representative microscopic images (20× magnification) of *in vitro* binding to immobilized BSA-mannose or BSA alone. Bacteria bound to the matrix were stained with crystal violet and the corresponding absorbance values are shown in Figure 2(a). D-mannose: 4-Aminophenil α-D-mannopyranoside.

		Residue position in FimH (unprocessed)								
		**43**	**74**	**89**	**126**	**131**	**137**	**157**	**245**	**317**
	**Consensus sequence**	**F**	**T**	**Q**	**L**	**Y**	**K**	**I**	**V**	**I**
**Strain identification code**	SL1344*	·	.	.	.	.	.	.	.	.
	B3589	**S**	.	.	.	.	.	.	.	.
	ATCC 14028	.	.	.	.	.	.	**F**	.	.
	NC983	.	.	.	.	.	.	**F**	.	.
	E40V	.	.	.	.	.	.	**F**	.	.
	Var. 5-CFSAN067216	.	.	.	.	.	.	.	**A**	.
	E2840153550	.	.	.	.	.	.	.	**A**	.
	SAP17-8290	.	.	.	.	.	.	.	**A**	.
	**IRTA GN-3728**	.	.	**R**	**R**	**S**	.	.	.	**N**
	Sg_wt7	.	.	**R**	**R**	**S**	.	.	.	**N**
	FORC88	·	**M**	**R**	**R**	**S**	**M**	.	.	**N**

Figure S5. Comparison of FimH amino acid sequences from *S.* Typhimurium. The consensus sequence, obtained after alignment using MUSCLE (v3.8.425), is shown at positions with polymorphisms. Residues identical to the consensus are indicated by dots. The wild-type strain IRTA GN-3728 is showed in bold. *, SL344 shares the FimH amino acid sequenced with the following strains: ST931R, CFSAN008081, 22792, var. 5-CFSAN067217, 10ST07093, 01ST04081, E40, 08-365-0802, RSE04, TJWQ005, SAP18-6199, SAP17-7699, SAP17-7399, STm1, STm6, STm10, STm11, STm12, LT2, ATCC 14028S, ST4/74, UK-1, 798, D23580 and A130.

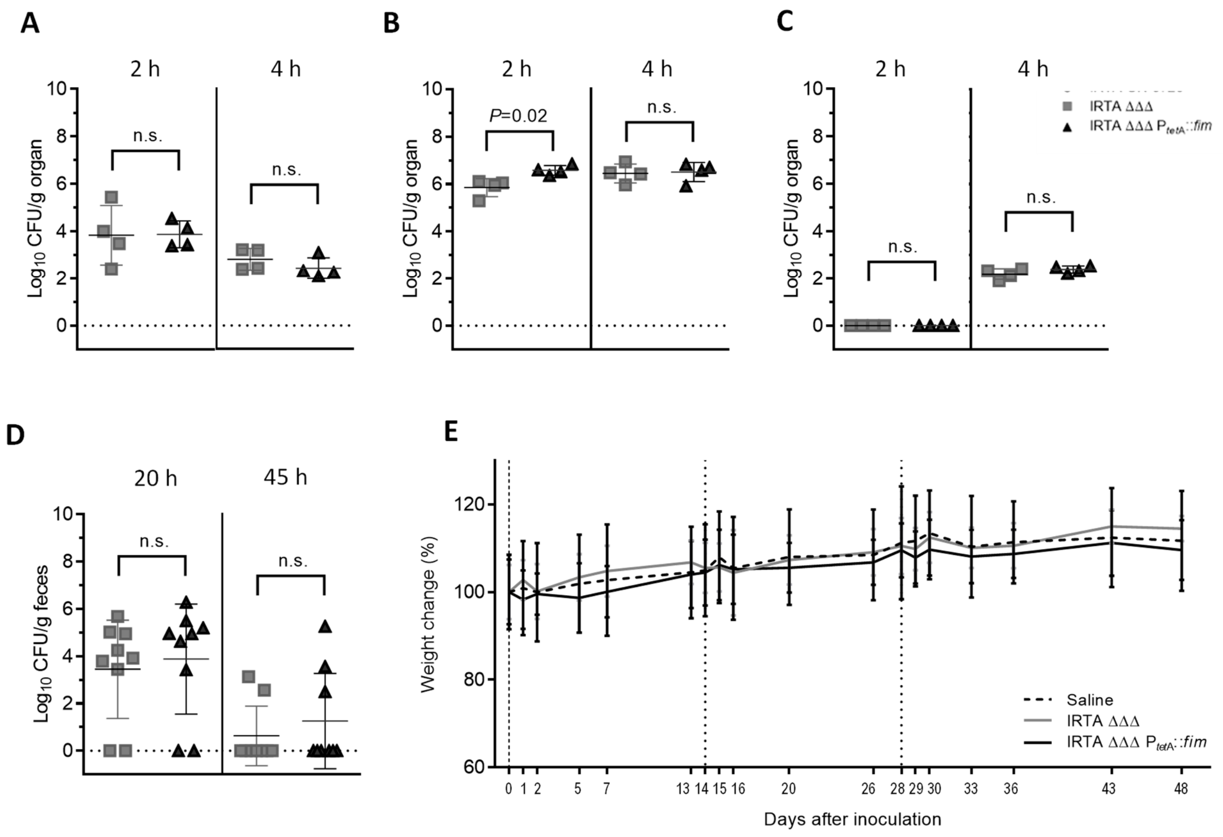

Figure S6. Safety profiles of *S*. Typhimurium auxotrophic derivatives. Bacterial loads in (A) small intestine, (B) large intestine plus cecum, (C) mLNs and (D) fresh fecal samples, recovered from BALB/c mice at the specified time points after inoculation with 10^9 CFUs of IRTA ΔΔΔ or IRTA ΔΔΔ P_{tetA}::kan (mean ± SD). Mann-Whitney *U* test. n.s., not significant. (E) Percentage change in body weight in inoculated mice relative to control mice (administered saline) (mean ± SD).

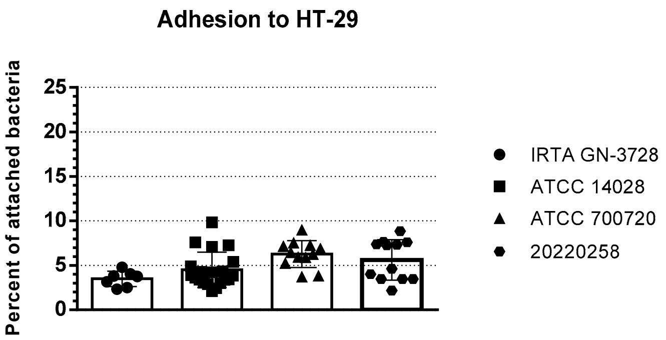

Figure S7. Percentage bacterial adherence to HT-29 colorectal cells (mean ± SD) obtained from *S*. Typhimurium cultures after growing under conditions inducing type 1 fimbriae (3rd static serial passage).

Table S1. Plasmids and primers used in this study.

Plasmid or primer	Relevant features or sequence (5'→3')	Reference
**Plasmid**		
p3773	Template plasmid derived from p2795	[1] [Prof. Michael Hensel, University of Osnabruck]
pKD46	Temperature sensitive replication (repA101ts) plasmid containing λ Red genes (*exo*, *bet*, *gam*) under arabinose-inducible promoter (P_{araB}) and an ampicillin resistant marker (*bla*$_{TEM-1}$)	[2]
**Primer**		
Ptet-PfimA-Fw (KORed mutagenesis)	GAAATGTTTAATTTATTACCGTGACGAAATGTCATATTCGGTACCGTGTAGGCTGGAGC	[1]
Ptet-PfimA-Rv (KORed mutagenesis)	GTTTCATGGATTTCCCTTGAATTACACACACCCGGTTTCGCATTACCTGGTTTTTTGATGC	[1]
PfimA-Fw (PCR and sequencing)	ATTCATCACCCTGGCTATGG	This study
PfimA-Fw2 (Sequencing)	GGAAGCCCTGCAAAGTAAAC	This study
PfimA-Rv (PCR and sequencing)	TTGATCGGCGGATTTAGTGC	This study
PfimA-R2seq (Sequencing)	AGAGCCAGCCTTCTTATTCG	This study
PfimA-94-left (qRT-PCR, UPL #94)	GCTTTCTCTGGTCAGGCAGA	This study
PfimA-94-right (qRT-PCR, UPL #94)	CCGGTTGCGGTAGTGCTATT	This study
rpoD-131-left (qRT-PCR, UPL #131)	CCTGATGCCGATGATCTGCT	This study
rpoD-131-right (qRT-PCR, UPL #131)	AGCAGCTTCTTCCGCATCTT	This study

Supplementary References

[1] Hansmeier N, Miskiewicz K, Elpers L, Liss V, Hensel M, Sterzenbach T. Functional expression of the entire adhesiome of *Salmonella enterica* serotype Typhimurium. Sci Rep. 2017;7(1):10326. doi: 10.1038/s41598-017-10598-2.

[2] Datsenko KA, Wanner BL. One-step inactivation of chromosomal genes in *Escherichia coli* K-12 using PCR products. Proc Natl Acad Sci U S A. 2000;97(12):6640-5. doi: 10.1073/pnas.120163297.
